# Hemorrhagic schwannoma of the trochlear nerve: Case report and a review of the literature

**DOI:** 10.3389/fonc.2022.1097155

**Published:** 2023-01-13

**Authors:** Jin Lei, Yu Li, Xueyan Wan, Junwen Wang, Chao You, Kai Zhao, Hongquan Niu

**Affiliations:** Department of Neurosurgery, Tongji Hospital, Tongji Medical College, Huazhong University of Science and Technology, Wuhan, China

**Keywords:** intracranial schwannoma, trochlear nerve, intratumoral hemorrhage, neurosurgical oncology, subtemporal approach

## Abstract

**Background:**

Schwannomas of the trochlear nerve with the absence of systemic neurofibromatosis are considerably uncommon, especially complicated by intra-tumoral hemorrhage. Due to the lack of typical clinical manifestations and imaging findings, a definite diagnosis of trochlear schwannomas before surgery is particularly difficult.

**Case presentation:**

We report the case of a 64-year-old female patient who presented with a unilaterally intermittent headache of 2-month duration and without a remarkable neurological deficit at admission. Imaging studies revealed a well-demarcated cystic-solid lesion with mixed signals beside the brainstem and suprasellar cisterna. The patient underwent a surgical operation with total resection of the tumor by a subtemporal surgical approach. The tumor was intraoperatively found to originate from the trochlear nerve and was pathologically confirmed as a hemorrhagic schwannoma with cystic degeneration.

**Conclusions:**

We describe this case in detail and conduct a concomitant survey of the literature, summarizing the clinical presentations, radiological features, surgical treatment, and the possible mechanisms of hemorrhage in relevance to trochlear nerve schwannoma.

## 1 Introduction

Cranial nerve schwannomas almost always arise from sensory nerves such as vestibular and trigeminal nerves (1). In very rare cases, they arise from motor nerves like oculomotor, abducens, and trochlear nerves and a majority of these cases occur in patients with systemic neurofibromatosis (2). Hence, motor-origin schwannomas such as the trochlear nerve schwannoma not secondary to neurofibromatosis are exceedingly rare. In addition, intratumoral hemorrhage from intracranial schwannomas is also infrequent. Since the first report in the year 1976 by King, just 43 surgical cases of trochlear nerve schwannomas that were confirmed by postoperative pathology have been reported in the English literature, of which only four were complicated with intratumoral hemorrhage (3–7). Here, we report an unusual case of hemorrhagic trochlear nerve schwannoma in a 64-year-old woman who presented with a unilaterally intermittent headache for 2 months. Intraoperatively, the trochlear nerve was found to be neoplastic and assimilated by the tumor and the tumor was consequently totally resected and pathologically confirmed to be hemorrhagic and cystic. Due to the rarity of trochlear nerve schwannoma, the previous literature was retrieved and summarized in terms of clinical symptoms, imaging features, and surgical treatment. Also, aspects with relevance to intra-tumoral hemorrhage were discussed. In this paper, the authors hope to provide additional references for the diagnosis and management of trochlear nerve schwannoma, especially when the tumor is complicated with acute hemorrhage.

## 2 Case presentation

**Figure 1:**
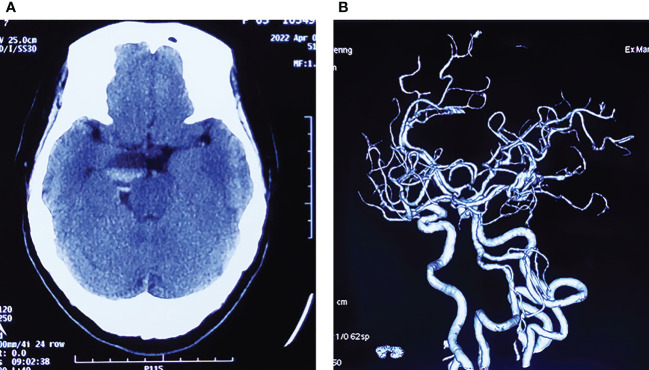
**(A)** Initial head CT scan showing an ovoid mass with mixed density located in the suprasellar cisterna, compressing the brain stem from the right side. **(B)** CT angiography suggests negative evidence of vascular lesions.

**Figure 2:**
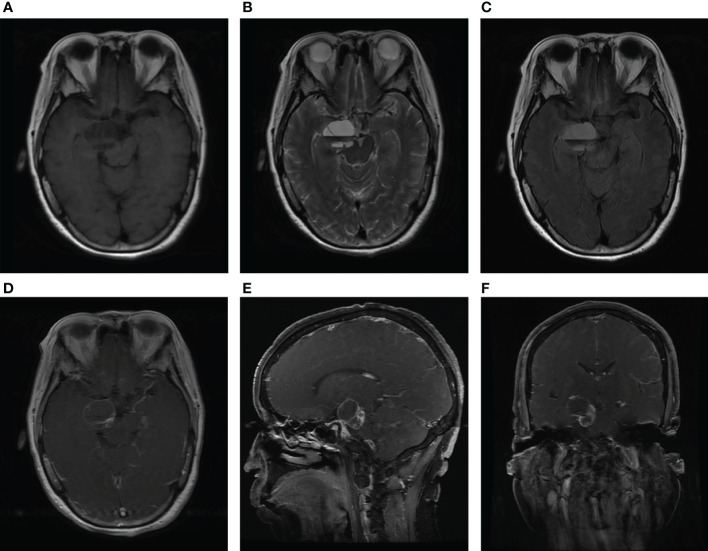
MRI scan at admission. Axial MRI without contrast showing the lesion with heterogeneous hypo-intense on T1-weighted images **(A)**, hyper-intense on T2-weighted images **(B)**, and hyper-intense on T2Flair images **(C)**. Axial **(D)**, sagittal **(E)**, and coronal **(F)** T1-weighted gadolinium-enhanced images show the heterogeneous contrast enhancement in the inner region of the mass, with a well-circumscribed enhanced cystic wall.

A 64-year-old female patient was admitted to our hospital due to a sudden onset headache on the right side 2 months ago with thereafter intermittent occurrence. The pain is with irregular patterns, occasionally accompanied by nausea. The patient had a 10-year history of hypertension and took antihypertensive drugs regularly with blood pressure controlled at a normal level. Unremarkable neurological deficits and no cutaneous signs of neurofibromatosis were found during the physical examination. Head computerized tomography (CT) scan, which was performed in the local hospital, showed an abnormal ovoid lesion with mixed density on the right side of the pons and suprasellar cisterna (**Figure 1A**). CT angiography suggested negative evidence of vascular lesions such as an intracranial aneurysm (**Figure 1B**). Magnetic resonance imaging (MRI) revealed the mass to appear heterogeneously hypo-intense on T1-weighted images, hyper-intense on T2-weighted images, and hyper-intense on T2Flair images. With a fluid level inside, the mass measured 29 mm in maximum diameter, compressing the pons and the midbrain from the right anterior direction (**Figures 2A-C**). Besides, the mass demonstrated heterogeneous contrast enhancement in the inner region with a well-circumscribed enhanced cystic wall after administration of the contrast agent gadolinium (**Figures 2D–F**). No other intracranial lesions were observed on the brain MRI. Laboratory data suggested unremarkable changes in blood routine tests, liver and renal function, and electrolyte levels. Tumor marker levels for germ cell tumors (lactic dehydrogenase, alpha-fetoprotein, or beta-hCG) were within the normal range.

**Figure 3:**
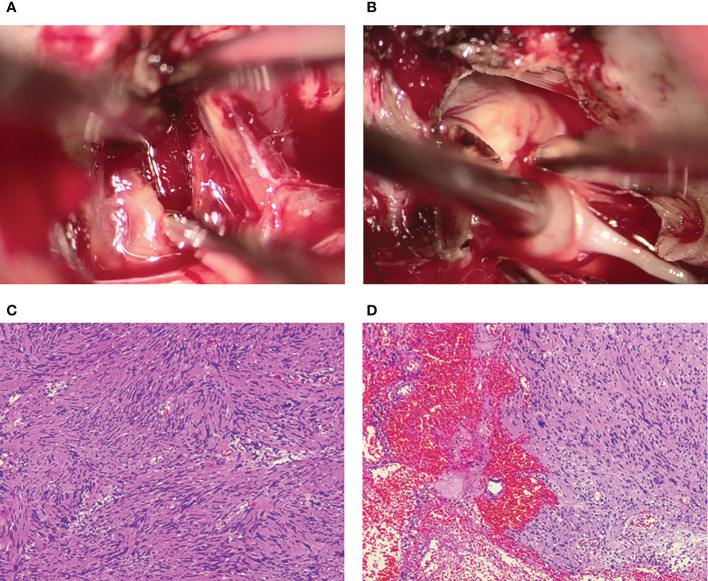
Intraoperative photographs show that the intratumoral hemorrhage is invisible after the incision of the tumor membrane **(A)**. The trochlear nerve tightly adheres to the tumor surface and is assimilated by the tumor **(B)**. Postoperative histopathology shows the characteristic features of a schwannoma complicated with intratumoral hemorrhage and cystic degeneration **(C, D)**.

After necessary preoperative preparation, the patient was brought to the operation room for resection of the mass. A subtemporal surgical approach was performed. Intraoperatively, a grey, cystic-solid tumor was encountered at the margin of the tentorium cerebellum, and the tumor went across the supratentorial and inferior tentorium. We split the tentorium cerebellum to expose the whole tumor and found it seriously adhered to the trochlear nerve. The tumor measured 23 ×20 ×12 mm in size. The trochlear nerve, without a clear boundary to the tumor, was partially neoplastic and assimilated by the tumor membrane. Based on the findings above, the diagnosis of a trochlear nerve-originated tumor was therefore made. Then the tumor membrane was incised for internal decompression and a large amount of hematoma was found inside the tumor. After the evacuation of the hematoma, the tumor was completely removed after amputation of the trochlear nerve and dissection from the surrounding tissues (**Figures 3A, B**). The tumor specimen was sent for pathological evaluation after surgery.

**Figure 4:**
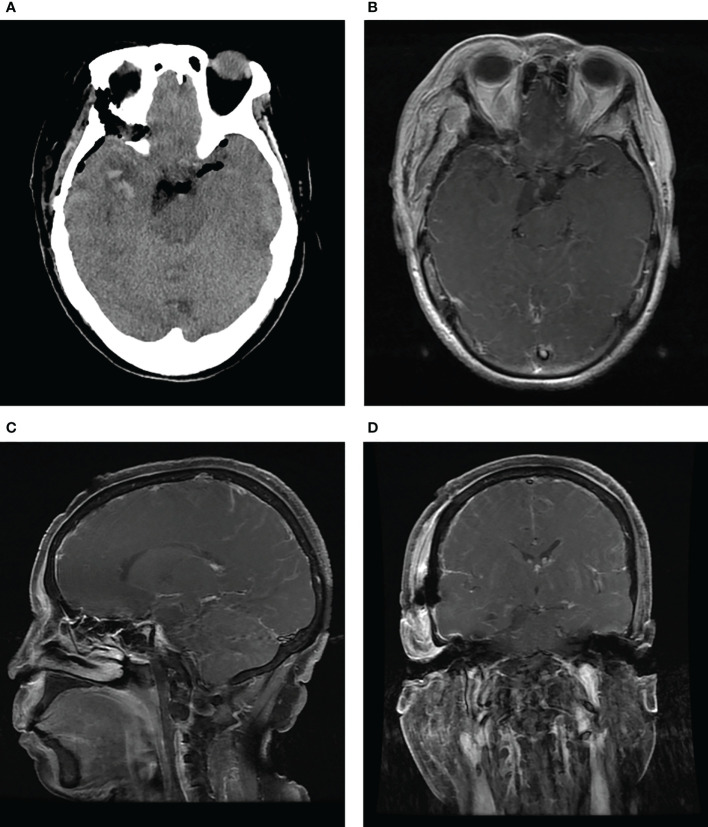
CT **(A)** and contrast-enhanced MRI **(B-D)** performed after surgery show total removal of the tumor and decompression of the brain stem.

Histopathological examination revealed the characteristic features of a schwannoma complicated with intra-tumoral hemorrhage and cystic degeneration (**Figures 3C, D**). The patient recovered uneventfully except for mild diplopia reported on the first day after surgery. Her headache disappeared, and the patient was discharged on the ninth postoperative day. Head CT and MRI performed postoperatively revealed total resection of the tumor (**Figure 4**). Four months after the tumor resection, the patient reported that her headache had completely resolved. The diplopia, although persisting as anticipated, had relieved spontaneously and did not have much impact on daily life.

## 3 Discussion

Schwannomas are typically benign, slow-growing nerve sheath neoplasms that can originate from both peripheral and cranial nerves (8). The cranial nerve schwannomas account for 8% of all intracranial tumors and develop in sensory nerves such as the vestibular nerve with the vast majority (9). Non-vestibular schwannomas, especially those arising from motor nerves such as the trochlear nerve, are extremely rare, in which the association with systemic neurofibromatosis has been previously established. This paper presents a very rare case of trochlear nerve schwannoma without neurofibromatosis, which was complicated by intratumoral hemorrhage. After radiological evaluation, the patient received microsurgical treatment with the tumor resected *via* the subtemporal approach. A hemorrhagic schwannoma of the trochlear nerve was confirmed by intraoperative findings and postoperative pathology.

Due to the rarity, an exhaustive literature review was performed based on the PubMed and Scopus databases using the following items: “trochlear nerve”, “schwannoma”, “neurinoma”, or “nerve sheath tumor” to identify the formerly published cases treated by surgery and with a pathological diagnosis. There were 43 surgical cases from 41 articles that met the criteria, including the one presented in our study (3–7, 10–45). A detailed description of demographical information, imaging features, and surgical treatment of all patients was summarized in **Supplemental Table 1**. The average age at presentation was 45.9 ± 16.2 years, with a range of 12–71 years and a peak of 51–55 years age range. There was no significant difference in gender with the female/male ratio 22:21. The initial symptoms last for an average of 9.7 months (range: 1 week to 5 years) and the patients were postoperatively followed up for 19.5 months average (range: 0–12 years).

According to our review, the initial symptoms for patients with trochlear schwannomas vary and manifest in a non-specific manner, including diplopia (n = 25; 58.1%), headache (n = 22; 51.1%), hemiparesis (n = 17; 39.5%), ataxia (n = 13; 30.2%), facial pain or paresis (n = 17; 39.5%), and hearing loss (n = 2; 4.6%). These extratrochlear symptoms may be explained by the tumor location, where the tumor commonly grows close to the tentorium and may compress the pons, the midbrain, and the adjacent cranial nerves (III, IV, V, VI, VII, or VIII) (46). As a relatively specific symptom, trochlear nerve palsy is only identified among 53.5% (n = 23) of all patients. One of the reasons may be that the impaired superior oblique function was well compensated for by other extraocular muscles among some patients, who did not complain of double vision at hospital visits (4). The other may be the relatively small tumor size in some patients since the presence of trochlear palsy has previously been reported to have an association with a tumor size larger than 30 mm (6). Therefore, for patients presenting with unilateral atypical neurologic symptoms rather than trochlear palsy, particularly if the imaging results highly suspect a trochlear nerve-origin tumor, the diagnosis of a schwannoma should not be missed.

Radiological imaging is the most utilized diagnostic technique in the evaluation of intracranial schwannomas. A typical schwannoma of the trochlear nerve on a head CT scan tends to appear as a single, well-demarcated, and solid lesion, located mainly in the course of the trochlear nerve and shows isointense or hypointense on T1-weighted, hyperintense on T2-weighted images, and intense enhancement after contrast administration at MRI (47). However, imaging features of such still lack adequate specificity. The differential diagnosis of an isolated, space-occupying lesion in the cisternal area adjacent to the brainstem should include a variety of neoplastic and vascular pathologies apart from a schwannoma, especially when the lesion is complicated with hemorrhage. Although uncommon, there were several cases reported to demonstrate imaging features similar to those of a trochlear nerve schwannoma, including meningioma (48), cavernous malformation (49), and thrombosed aneurysms (50). Hence, intraoperative findings and histopathological examination are indispensable to the definitive diagnosis of a trochlear nerve schwannoma.

Trochlear nerve schwannomas were classified by Celli et al. mainly into three types based on the tumor location: cisternal, cisternocavernous, and cavernous types (16). Cisternal trochlear schwannomas were found in most of the reported cases (n = 34; 79.1%), whereas the cisternocavernous (n = 4; 9.3%) and cavernous types (2/43; 4.6%) were less observed. Intriguingly, in our review, there are another three cases in which trochlear nerve schwannomas developed in the pineal region (37, 39, 43). The region of the pineal gland is not a usual location for trochlear schwannomas. Al-Hussaini et al. described, in their cohort of 633 patients, that germ cell tumors account for 59% of all pineal neoplasms, followed by pineal parenchymal tumors (30%), gliomas (5%), atypical rhabdoid/teratoid tumor (<1%), and others (6%; e.g., pineal cysts, lymphomas, papillomas, vascular malformations, etc.) based on the histologic subtypes (51). Thus, as a differential diagnosis for trochlear schwannomas with their origin in this region, the possibility of the above should at least be considered.

The standardized guidelines for the management of trochlear nerve schwannomas have not yet been established (52). However, according to a recent expert consensus on non-vestibular schwannomas, therapeutic options include clinical observation through regular imaging follow-up, stereotactic radiosurgery (SRS), or microsurgical resection (53). Generally, for patients with a small schwannoma that presents with no symptoms or with diplopia only, observation with sequential MR scans may be a reasonable choice, because trochlear nerve schwannoma grows very slowly, with an estimated average of 0.19 mm per year (54). Clinical observation is also recommended for the elderly or patients with surgical contraindications. Once the tumor size increases obviously during the follow-up period, radiosurgery or surgical intervention needs to be considered, depending on the tumor size. Rapid tumor growth in the short-term or acute exacerbation of clinical symptoms usually indicates the occurrence of cystic degeneration or intratumoral bleeding, which require surgery as soon.

According to the review, various approaches were used for patients who received tumor resection, such as transtemporal subtemporal (n = 15), lateral suboccipital (n = 10), transpetrosal (n = 7), and pterional (n = 5) approaches. The patients all have a good outcome with total (n=36; 83.7%) or at least subtotal (n = 7; 16.3%) removal of the tumor. The selection of a certain approach is mainly based on the direction of tumor growth and the preference of the surgeon. In our patient, the classical subtemporal approach was used as in most prior cases. With this approach, adequate brain relaxation was achieved *via* spinal cerebrospinal fluid drainage, and then the temporal lobe was elevated after the division of several draining veins on the inferior surface of the brain tissue (55). The tumor was able to be completely excised with the protection of the surrounding vital structures *via* this approach. On the other hand, the lateral suboccipital approach seems to gain more popularity in recent years. The rationale for this approach may be that a large portion of trochlear schwannomas originates from the infratentorial region. Further, the suboccipital approach is associated with a lower incidence of intraoperative complications such as temporal lobe contusion or injury to large cerebral veins, for example, the Labbé vein (45).

Our review showed that trochlear nerve palsy occurred in 76.1% (n = 32) of the patients during the postoperative follow-up. As the origin nerve of schwannomas, it is inseparable from the tumor intraoperatively. In addition, the trochlear nerve is the longest and thinnest among all cranial nerves, which made it prone to injury. Thus, the trochlear nerve would be hardly kept anatomically intact intraoperatively and tends to be sacrificed in the removal of the tumor (4). However, even without preservation of the trochlear nerve, some patients seemed to behave well because the impaired nerve function could be partially compensated for by other extraocular muscles. Therefore, although there is still debate about whether to preserve the nerve *via* retaining the tumor capsule or to repair the disrupted nerve, total resection of the tumor, if possible, is recommended by most scholars (32, 44, 45).

Intratumoral hemorrhage is not uncommon for intracranial malignancies such as high-degree gliomas or metastatic tumors, and some benign tumors like large meningiomas. However, hemorrhage occurring in trochlear schwannomas is rarely encountered with only four cases reported to date. Of all four patients, acute onset of symptoms and signs was observed, including significantly increased intracranial pressure (i.e., headache, nausea, vomiting, and double vision) (7), acute exacerbation of the original symptoms (i.e., left hemiparesis and right trochlear paralysis) (6), persistent hiccup (5), or sudden onset of diplopia (4). The duration time between the acute onset of symptoms and admission was relatively short, ranging from 10 days to 3 weeks. In our case, the sudden headache occurring 2 months before admission may be caused by intratumoral hemorrhage, rapidly increasing the tumor volume and generating compression to the surrounding brain tissue. Our speculation could be supported by the CT/MRI scan at admission, which suggested that the hematoma was partially liquefied with a fluid level inside the tumor.

The pathogenesis of spontaneous hemorrhage from schwannoma is not fully understood. As with all tumors, rapid tumor expansion and inadequate blood supply may cause central necrosis, cystic degeneration, and consequent hemorrhage (56). The trochlear schwannoma of our patient may follow this course because the postoperative pathology confirmed the existence of cystic degeneration and intratumoral hemorrhage within the tumor specimen. On a microscopic level, cystic schwannomas are relatively vascular with the proliferation of abnormally dilated and thin-walled microvessels which may undergo a spontaneous thrombosis or rupture and thus cause intratumoral hemorrhage (57). Several studies on vestibular schwannoma also reported additional contributing factors for bleeding, including the use of anticoagulation drugs (58), trauma (59), and tumor larger than 25 mm in size (57). Therefore, the exact mechanisms for schwannoma hemorrhage are likely to be multifactorial and certainly warrant further research.

## 4 Conclusions

Trochlear nerve schwannomas are rarely encountered worldwide with only a limited number of cases having been reported over the past decades. Due to the lack of specific symptoms and radiological features, a definite preoperative diagnosis remains a challenge. The therapeutic regimen includes clinical observation, stereotactic radiology, and surgical resection and needs to be individualized for each patient. Total resection can be achieved through various approaches, but trochlear nerve palsy is a common postoperative complication. Moreover, the potential mechanisms of intratumoral hemorrhage occurring in schwannomas may be multifactorial and need further research.

## Data availability statement

The raw data supporting the conclusions of this article will be made available by the authors, without undue reservation.

## Ethics statement

Ethical review and approval was not required for the study on human participants in accordance with the local legislation and institutional requirements. The patients/participants provided their written informed consent to participate in this study.

## Author contributions

All authors listed have made a substantial, direct, and intellectual contribution to the work and approved it for publication.
